# Pulmonary metastasis of giant cell tumor of bones

**DOI:** 10.1186/1477-7819-12-261

**Published:** 2014-08-20

**Authors:** Aikeremujiang Muheremu, Xiaohui Niu

**Affiliations:** Department of Orthopedic Oncology Surgery, Beijing Ji Shui Tan Hospital, 31 Xinjiekou East Street, 100035 Beijing, Xicheng District China

**Keywords:** Giant cell tumor, Bone, Pulmonary metastasis

## Abstract

Giant cell tumor of bone (GCTB) accounts for 5% of primary skeletal tumors. Although it is considered to be a benign lesion, there are still incidences of pulmonary metastasis. Pulmonary metastasis of GCTB may be affected by tumor grading and localization as well as the age, gender and overall health status of the patient. Patients with local recurrence are more likely to develop pulmonary metastasis of GCTB. High expression of some genes, cytokines and chemokines may also be closely related to the metastatic potential and prognosis of GCTB. The treatment of the primary GCTB is key to the final outcome of the disease, as intralesional curettage has a significantly higher local recurrence and pulmonary metastasis rate than wide resection. However, even patients with pulmonary metastasis seem to have a good prognosis after timely and appropriate surgical resection. It is hoped that with the development of novel surgical methods and drugs, pulmonary metastasis of GCTB can be prevented and treated more effectively.

## Review

Giant cell tumor of bone (GCTB) is a primary intramedullary tumor which is supposedly benign but can be locally aggressive and even metastatic [1–3]. Its name originated from the giant cells found within the tumor. Those giant cells share specific markers with osteoclasts such as tartrate-resistant acid phosphatase [4], cathepsin K [5], carbonic anhydrase II [6], calcitonin receptor [7] and receptor activator of nuclear factor-κB (RANK) [8]. They both have bone resorption capabilities, but giant cells are significantly larger than the osteoclasts and have hundreds of nuclei [9].

GCTB account for 5% of primary skeletal tumors and 21% of all benign bone tumors [10, 11]. It has the highest prevalence among the population aged between 20 and 40-years-old and has a slight female predominance [12], with the female to male ratio of 1.3 to 1.5:1.0 [13]. The most usual sites for GCT include distal femur, proximal tibia and distal radius [14–18]. The incidence of GCTs in small bones is less than 1% of all GCT cases [19, 20].

## Diagnosis of giant cell tumor of bone

Conventional diagnostic methods of GCTB include its clinical, radiological and histopathological manifestations. Diagnostically, typical clinical manifestations of GCTB include regional pain, swelling and occasionally history of pathological fracture [21]. Radiographs demonstrate an eccentric lytic lesion with cortical extension. Histologically, GCT consists of multiple multinucleated giant cells with strong phenotypic similarities to osteoclasts and spindle-shaped proliferative stromal cells [15, 22].

More recently, the increasingly popular application of novel imaging techniques such as positron emission tomography/computerized tomography (PET/CT) has made the diagnosis of primary and metastatic GCTB more accurate [23]. Furthermore, basic research has also provided diagnostic gene markers such as P53, P63 and P73 [24, 25], as well as KTN 1, NEB, ROCK1 and ZAK [26]. However, more research is needed before these biomarkers can be used in clinical practice.

## Pulmonary metastasis of giant cell tumor of bone

### Prevalence

Although rare, GCTB can be multicentric or arise in locations where it is difficult to be surgically removed. The course of progression of GCTB varies and can range from local bony destruction to local metastasis. The distant metastasis and malignant transformation are extremely rare conditions [27]. Rare cases of metastases to other sites have been described in the literature. Those reported include the lung, lymph nodes [28], liver, soft tissue [29], brain, mediastinum, scalp, kidney and penis.

Based on the current literature, approximately 3% of GCTB metastasizes to the lung (Table 1). Pulmonary metastasis is very rarely discovered during diagnosis of the initial GCTB, but more frequently in cases of recurrence. The interval from the time of surgery on the primary lesion to the occurrence of pulmonary metastasis can be as little as several months or more than 10 years. However, in most cases metastases were found within three years after the surgical treatment of the initial lesion [30, 31]. Based on the current literature (Table 1), approximately 3% of GCTB metastasizes to lung at certain time points after the confirmed diagnosis of primary GCTB.

**Table 1:** **Some studies on pulmonary metastasis of giant cell tumors of bone**

Study (Author/year)	Time of follow up	Number of patients	Ratio of pulmonary metastasis
Errani *et al*. 2010 [32]	91 (36-204) months	349	4%
Kremen *et al*. 2012 [33]	0.1-312 months	230	2%
Takeuchi *et al*. 2011 [34]	2-180 months	110	7.5%
Klenke *et al*. 2011 [35]	108 (36-233)months	118	4.2%
Viswanathan *et al*. 2010 [36]	4 months-12 years	470	1.7
Balke *et al*. 2008 [37]	60 (8-280) months	214	3.3%
Donthineni *et al*. 2006 [38]	18-126months	51	13.7%
Faisham *et al*. 2006 [39]	12-60 months	20	30%
Dominkus *et al*. 2006 [30]	3 months to 11.9 years	649	2.1%
Prosser *et al*. 2005 [40]	70 (24-214) months	137	1.6%
Su *et al*. 2004 [41]	62 (28-138) months	87	1.2%
Trieb *et al*. 2001 [42]	11 (4-43) years	47	0%
Blackley *et al*. 1999 [43]	80 (29-132) months	59	1.7%
O’Donnell *et al*. 1994 [44]	4 (2-10) years	60	0%
Kay *et al*. 1994 [45]	23-120 months	66	9.1%
Campanacci 1 *et al*. 987 [1]	2-44 years	280	2.1%
McDonald *et al*. 1986 [46]	84 (min 24) months	146	3.2%

### Factors related to the pulmonary metastasis of giant cell tumor of bone

There is no consensus on which factors may affect the likelihood of pulmonary metastasis of GCTB. However, several independent studies on some special types of GCTB may bring some insights into this issue. For example, Faisham *et al*. [39] have reported a pulmonary metastasis ratio of 30% in patients with grade 3 GCTB by Campanacci’s classification, indicating that the tumor grading might be an important prognostic factor for the prediction of pulmonary metastasis of GCTB. Donthineni *et al*. [38] have reported a pulmonary metastasis ratio of 13.7% in patients with GCTB which occur primarily on spine, and Errani *et al*. [32] reported a higher incidence of pulmonary metastasis of GCTB located in proximal femur and distal radius, indicating that the location of primary lesion might also be a prognostic factor. However, most authors believe that there is no significant relationship between the location of GCTB and its risk of pulmonary metastasis.

Rock *et al*. [47] had reported a six-fold higher risk of lung metastasis in patients who experienced local recurrence of GCTB compared with patients with no history of local recurrence. Studies by Rock and several other authors indicate that higher risk patients with recurrent GCTB should be carefully monitored for pulmonary metastases after the appropriate treatment of the recurrent lesion (Table 2). Most studies assume that local recurrence of GCTB is a risk factor for lung metastasis.

**Table 2:** **Studies involving the relationship between local recurrence and pulmonary metastasis of GCTB**

Study (Author/year)	Number of patients	Number of patients with pulmonary metastasis	Local recurrence
Kremen *et al*. 2012 [33]	230	5	28
Klenke *et al*. 2011 [35]	118	5	24
Errani *et al*. 2010 [32]	349	14	52
Prosser *et al*. 2005 [40]	137	22	26
Trieb *et al*. 2001 [42]	47	0	11
Blackley *et al*. 1999 [43]	59	10	12
Kay *et al*. 1994 [45]	66	6	18
O’Donnell *et al*. 1994 [44]	60	0	15
Viswanathan *et al*. 2010 [36]	470	8	13 of 24 patients with lung metastasis
Tubb *et al*. 1992 [48]			7 of 13 patients with lung metastasis
Goldenberg *et al*. 1970 [28]	218	6	5 of 6 patients with lung metastasis

Moreover, literature of basic research has also provided us with methods to predict the possibility of pulmonary metastasis. For example, pro-osteoclastogenic cytokines such as receptor activator of nuclear factor kappa-B ligand (RANKL), interleukin (IL)-6 and tumor necrosis factor (TNF), as well as monocyte-recruiting chemokines such as stromal cell-derived factor-1 (SDF-1) and monocyte chemoattractant protein (MCP)-1, participate in unfavorable osteoclastogenesis and bone destruction [49]. High expression of those cytokines and chemokines may help predicting the metastatic potential and prognosis of GCTB.

### Treatment

Treatment of giant cell tumor of bone is still one of the most controversial and discussed issues [50]. The course of progression of metastatic pulmonary disease has been found to be as unpredictable as the primary tumor [51]. Since the incidence of pulmonary metastasis of GCTB is rather rare, there is no study on the systematic treatment of metastatic GCTB in the current literature. However, since the recurrence rate of GCTB is significantly related to its pulmonary metastasis, studies concerning treatment methods and the recurrence rate of GCTB may provide guidance for the prevention of metastasis and achieve a better prognosis.

### Surgical treatment

Surgical treatment options for primary GCTB lesions include intralesional surgery and wide resection. Intralesional surgery has a higher recurrence rate but does preserve adjacent joint function. On the other hand, although wide resection may prevent metastasis, it may seriously impair the function of adjacent joints. When curettage is performed, local adjuvants such as polymethylmethacrylate (PMMA), phenol, alcohol, cryotherapy and hydrogen peroxide have been reported to significantly decrease the rate of local recurrence and pulmonary metastasis [10, 11, 38, 52, 53]. However, some other studies [40, 54] failed to find any positive therapeutic effect of these adjacent measures. In a Scandinavian Sarcoma Group multicentric study by Kivioja *et al*. [11] involving 294 patients, the recurrence rate of GCTB was 20% in patients treated with bone cement, which is significantly lower (*P* = 0.001) than the 56% recurrence rate in patients who underwent intralesional surgery with bone grafts. On the contrary, in a Canadian multicentric study of 186 patients by Turcotte *et al*. [55], filling the remnant cavity with bone cement did not significantly reduce the recurrence rate of GCTB (Table 3).

**Table 3:** **Recurrence rates after different surgical treatment of giant cell tumors of bone**

Study (Author/year)	Number of patients	Wide resection	Intralesional curettage	Intralesional curettage + regional burring	Intralesional curettage + PMMA
Klenke *et al*. 2011 [35]	118	5	N/A	32	+ phenol + burrin:15
Errani *et al*. 2010 [32]	349	12	N/A	18	
Balke *et al*. 2008 [37]	214	0	140	47	+phenol: 38
Kivioja *et al*. 2008 [11]	294	12	51	N/A	22
Knochentumoren 2008 [52]	256	2	49	N/A	+phenol:69
Malek *et al*. 2006 [56]	40	N/A	N/A	33	N/A
McGough *et al*. 2005 [57]	183	N/A	46	N/A	N/A
Prosser *et al*. 2005 [40]	137	0	N/A	N/A	+burring: 21
Su *et al*. 2004 [41]	87	3	N/A	N/A	+phenol: 18
Turcotte *et al*. 2002 [55]	186	34	N/A	N/A	+phenol + burring:55
Blackley *et al*. 1999 [43]	59	N/A	N/A	12	N/A
Campanacci *et al*. 1987 [1]	280	0	27	N/A	8
McDonald 1986 [45]	146	7	N/A	34	N/A

Results from the current literature implies that the recurrence ratio of GCT is: Intralesional curettage > Intralesional curettage + regional burring > Intralesional curettage + local adjuvants + PMMA > Wide resection.

Moreover, Enneking’s and Campanacci’s classifications [2, 58] are helpful tools for planning the initial surgical treatment. For instance, stage III GCTB is best managed with wide resection for better local control. Faisham *et al*. [39] proposed that aggressive treatment of pulmonary metastasis is mandatory in the management of aggressive GCTB. Surgical excision of solitary and surgically-accessible lesions as well as lung metastases is now widely accepted as the treatment of choice, with an acceptable long-term survival rate [45].

### Drug treatment

The anti-osteoclastic effects of bisphosphonates, along with their ability to protect bone from further resorption, make bisphosphonates potential candidates for the treatment of GCTB, and studies have confirmed their efficacy [59]. Balke *et al*. [37] examined clinical and radiological outcomes of treatment with amino bisphosphonates on 25 cases of aggressive primary, recurrent and metastatic GCTB derived from four European centers. Most inoperable sacral or pelvic tumors did not increase in size and no further recurrence or metastasis was observed in patients with recurrent GCTB. Lung metastases did not increase in size or number following treatment, suggesting that bisphosphonates may be useful against the progression of metastatic GCTB lesion in lung.

Denosumab, a fully human monoclonal antibody that specifically inhibits normal and tumor-associated bone lysis by preventing RANKL-mediated formation and activation of multinucleated osteoclasts or giant cells from RANK-positive mono-nuclear preosteoclasts and macrophages [21], has been proved to be able to reduce the number of RANK-positive giant cells and proliferative stromal cells [15]. In the study of Thomas *et al*. [60], 30 out of 35 (86%; 95% CI 70 to 95) of evaluable patients had a positive tumor response after the treatment of denosumab. It is likely a novel and effective method with great potential in the treatment of GCTB [61].

### Prognosis

**Figure 1:**
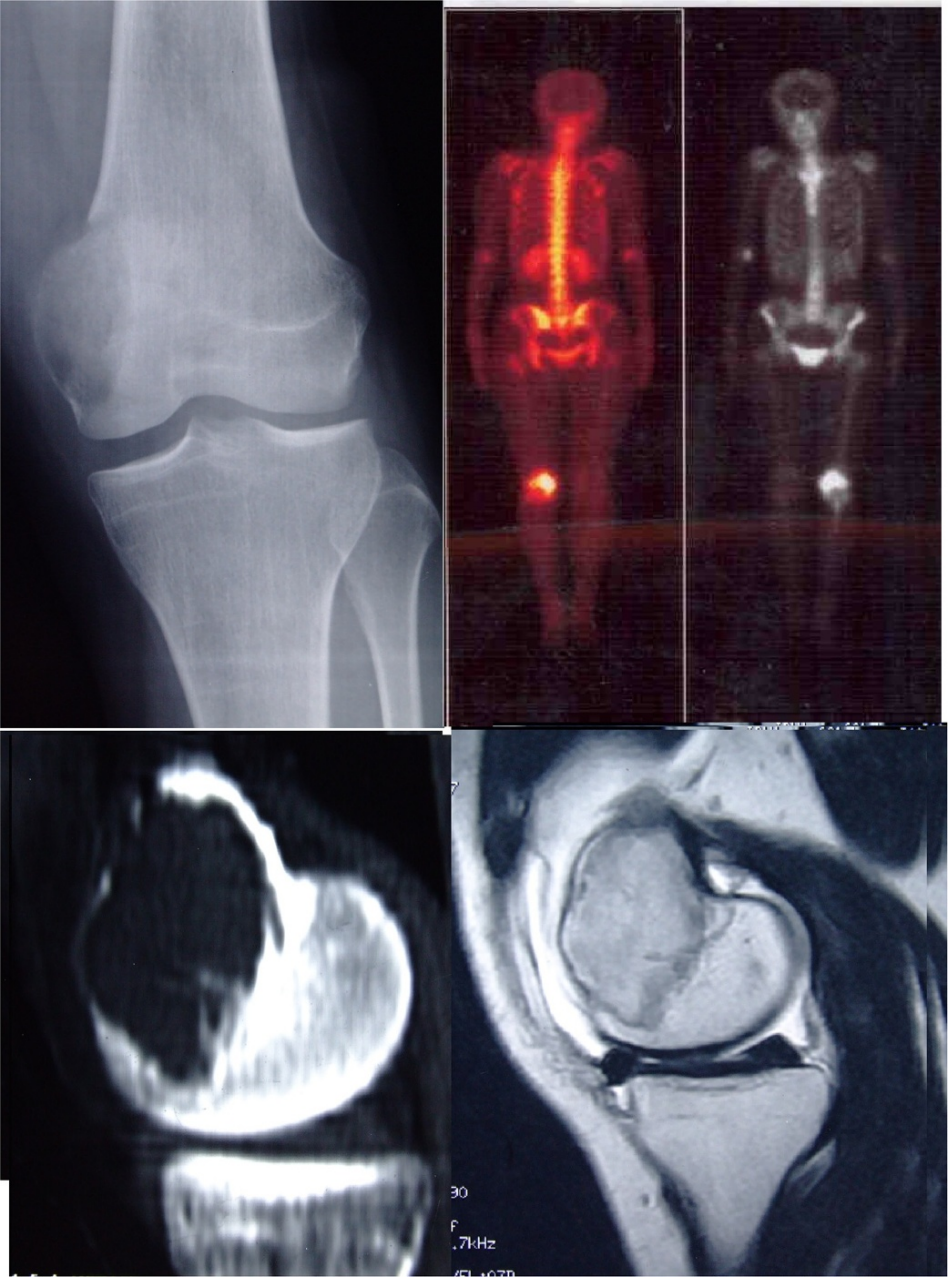
**A 22-year-old female patient came to the orthopedic clinic complaining of continuous pain of left knee, X-ray,CT, MRI and bone scan tests found a sub-articular lucent lesion with clear boundaries on the left distal femoral bone.**

**Figure 2:**
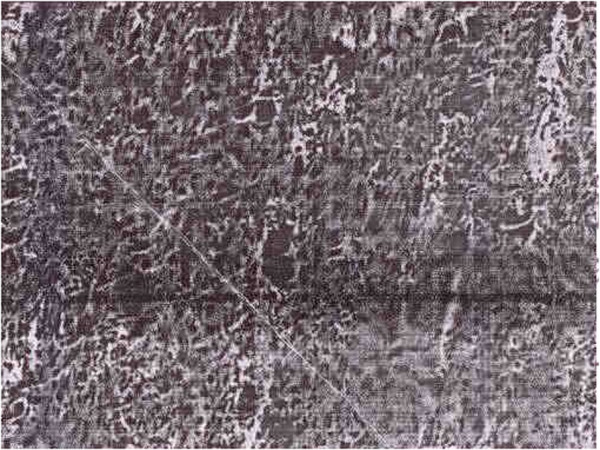
**Pathological examination was fine needle biopsy confirmed it was giant cell tumor.** Local curettage and bone grafting was performed by the local hospital.

**Figure 3:**
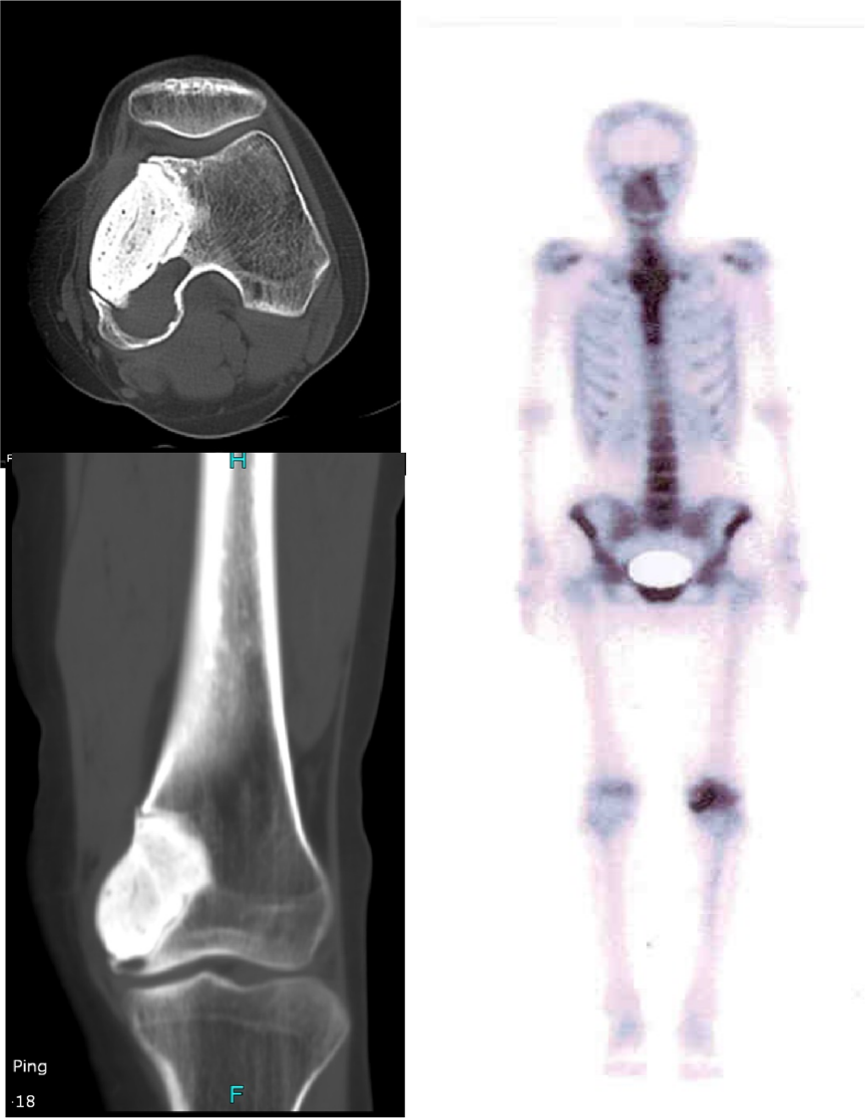
**18 months after the first surgery, CT and bone scan found local recurrence was found at follow-up visit.**

**Figure 4:**
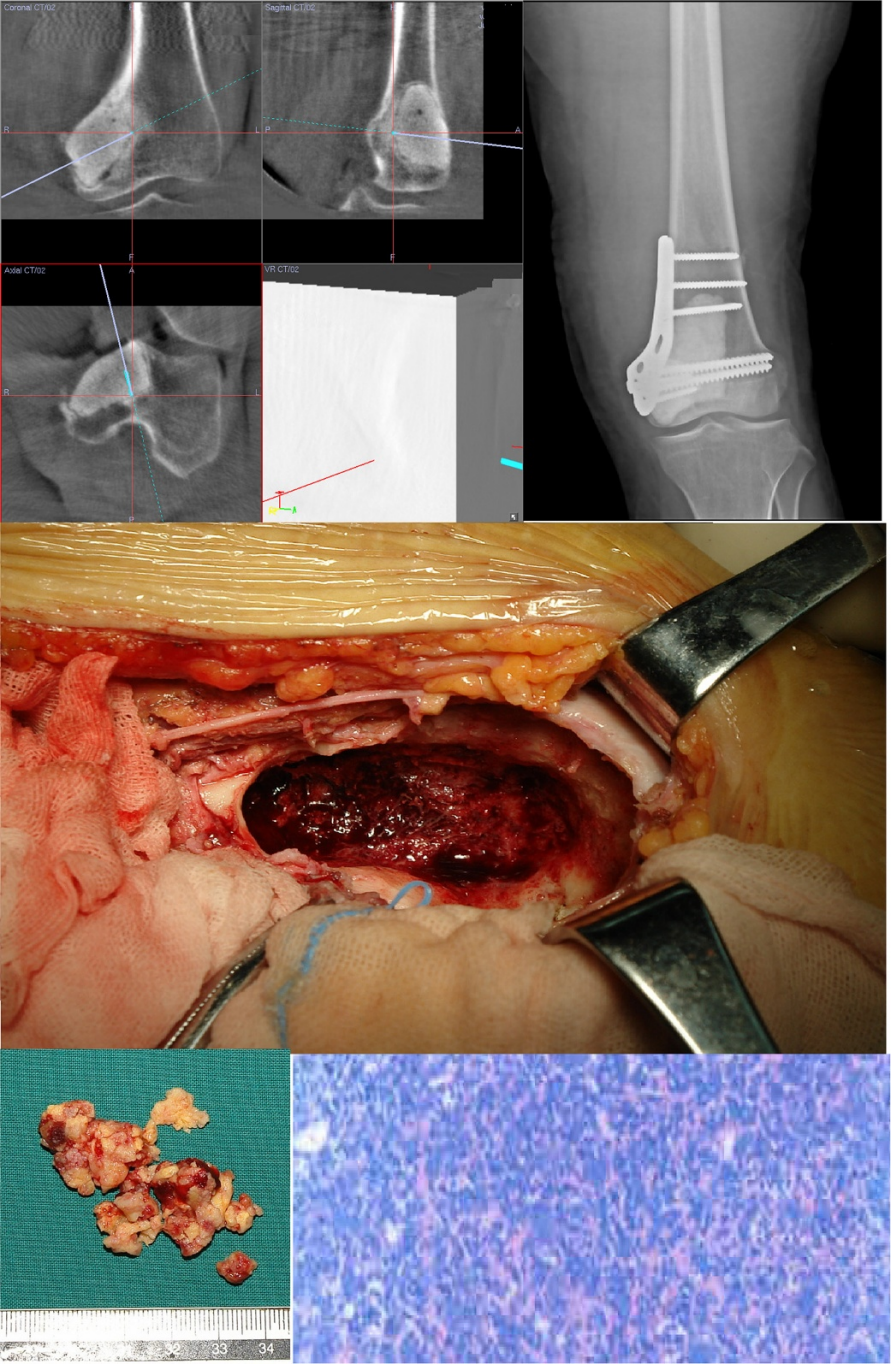
**She was treated with** ***en bloc*** **resection under the aid of computer navigation under intraoperative CT scan, and the cavity was filled with bone cement.** Intraoperative pathological exam confirmed the recurrence of GCTB.

**Figure 5:**
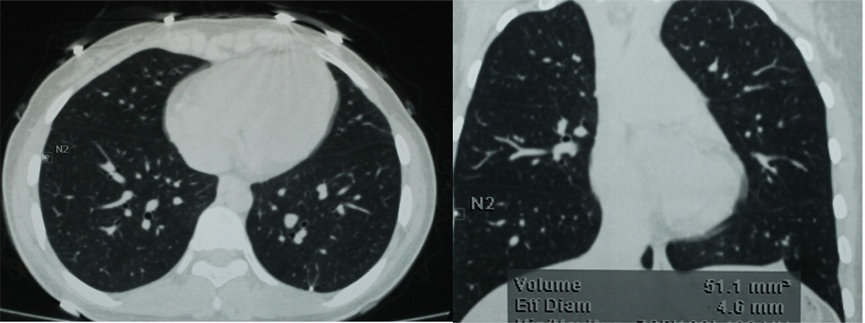
**34 months after the first surgery, multiple nodules were found by CT scan in both of her lungs on a follow-up visit.**

**Figure 6:**
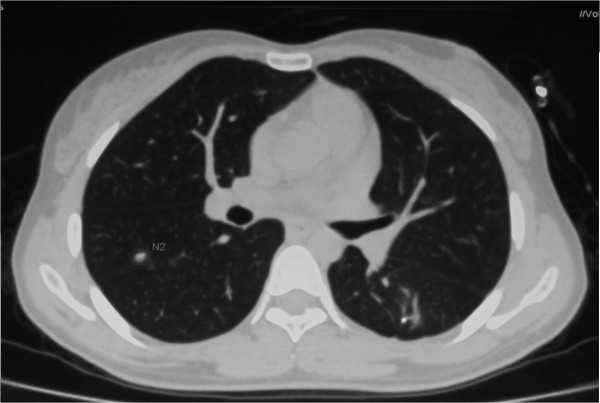
**At the last follow-up visit 40 months after the first surgery, Ct scan showed that the patient was alive with the disease, complaining of no trouble with breathing.**

For lung metastasis, appropriate surgical resection such as metastasectomy, wedge resection or lobectomy should be carried out if it is possible to prevent progressive pulmonary dysfunction [30, 62]. Because pulmonary metastases are rare and timely surgical and chemotherapeutic treatment is usually successful (Table 4, see Figures 1, 2, 3, 4, 5 and 6 for an example case), we believe the potential for metastases should not by itself be an indication for wide resection of primary tumors.

**Table 4:** **Number of patients with the incidence of pulmonary metastasis of giant cell tumors of bone**

Study (Author/year)	Number of patients	Cases with pulmonary metastasis	Survivors at the last follow up
Kremen *et al*. 2012 [33]	230	5	4
Takeuchi *et al*. 2011 [34]	110	8	8
Errani *et al*. 2010 [32]	349	14	10*
Klenke *et al*. 2011 [35]	118	5	4
Viswanathan *et al*. 2010 [36]	470	8	8
Faisham *et al*. 2006 [39]	20	6	5
Dominkus *et al*. 2006 [30]	649	14	14
Donthineni *et al*. 2006 [38]	51	7	5
Kay *et al*. 1994 [45]	7	7	6

*3 of the 4 patients died of causes irrelevant to GCTB.

## Conclusions

In conclusion, GCTB is normally considered a benign bone tumor. However, it may occasionally metastasize to vital organs such as the lung. The prognosis can be successful with timely and appropriate surgical treatment and chemotherapy. With the development of novel surgical methods and application of new drugs, most patients with GCTB can still achieve successful treatment results.

## Abbreviations

CT: computerized tomography
GCTB: Giant cell tumor of bone
IL: interleukin
MRI: magnetic resonance imaging
MCP: monocyte chemoattractant protein
PMMA: polymethylmethacrylate
PET/CT: positron emission tomography/computerized tomography
RANK: receptor activator of nuclear factor-κB
RANKL: receptor activator of nuclear factor kappa-B ligand
SDF: stromal cell-derived factor-1
TNF: tumor necrosis factor.

## References

[1] Campanacci M, Baldini N, Boriani S, Sudanese A (1987). Giant-cell tumor of bone. J Bone Joint Surg Am.

[2] Enneking WF (1983). Musculoskeletal Tumor Surgery.

[3] Feigenberg SJ, Marcus RB, Zlotecki RA, Scarborough MT, Enneking WF (2002). Whole-lung radiotherapy for giant cell tumors of bone with pulmonary metastases. Clin Orthop Relat Res.

[4] Anazawa U, Hanaoka H, Shiraishi T, Morioka H, Morii T, Toyama Y (2006). Similarities between giant cell tumor of bone, giant cell tumor of tendon sheath, and pigmented villonodular synovitis concerning ultrastructural cytochemical features of multinucleated giant cells and mononuclear stromal cells. Ultrastruct Pathol.

[5] Lindeman JH, Hanemaai jer R, Mulder A, Dijkstra PD, Szuhai K, Bromme D (2004). Cathepsin K is the principal proteas e in giant cell tumor of bone. Am J Pathol.

[6] Kotake S, Sato K, Kim KJ, Takahashi N, Udagawa N, Nakamura I (1996). Interleukin-6 and soluble interleukin-6 receptors in the synovialfluids from rheumatoid arthritis patients are responsible for osteoclast-like cell formation. J Bone Miner Res.

[7] Collier FM, Huang WH, Holloway WR, Hodge JM, Gillespie MT, Daniels LL (1998). Osteoclasts from human giant cell tumors of bone lack estrogen receptors. Endocrinology.

[8] Atkins GJ, Kostakis P, Vincent C, Farrugia AN, Houchins JP, Findlay DM, Evdokiou A, Zannettino AC (2006). RANK expression as a cell surface marker of human osteoclast precursors in peripheral blood, bone marrow, and giant cell tumors of bone. J Bone Miner Res.

[9] Balke M, Campanacci L, Gebert C, Picci P, Gibbons M, Taylor R, Hogendoorn P, Kroep J, Wass J, Athanasou N (2010). Bisphosphonate treatment of aggressive primary, recurrent and metastatic giant cell tumour of bone. BMC Cancer.

[10] Lackman RD, Hosalkar HS, Ogilvie CM, Torbert JT, Fox EJ (2005). Intralesional curettage for grades II and III giant cell tumors of bone. Clin Orthop Relat Res.

[11] Kivioja AH, Blomqvist C, Hietaniemi K, Trovik C, Walloe A, Bauer HC, Jorgensen PH, Bergh P, Follerås G (2008). Cement is recommended in intralesional surgery of giant cell tumors: a Scandinavian Sarcoma Group study of 294 patients followed for a median time of 5 years. Acta Orthop.

[12] Harness NG, Mankin HJ (2003). Giant-cell tumor of the distal forearm. J Hand Surg.

[13] Jin CM, So RK, Myung JC, Yong CL (2012). Multiple pulmonary metastases from giant cell tumor of a hand. Am J Med Sci.

[14] Huvos AG, Huvos AG (1991). Tumors of histiocytic or fibrohistiocytic origin: giant cell tumor of bone. Bone Tumors, Diagnosis, Treatment, and Prognosis.

[15] Kim TS, Park JS (2001). Metastasising recurrent giant cell tumor: a case report. J Korean Bone Joint Tumor Soc.

[16] Mirra JM, Mirra JM (1989). Giant cell tumors. Bone Tumors; Clinical, Radiological, and Pathologic Correlations.

[17] Osaka S, Sugita H, Osaka E, Yoshida Y, Ryu J, Hemmi A, Suzuki K (2004). Clinical and immunohistochemical characteristics of benign giant cell tumour of bone with pulmonary metastases: case series. Orthop Surg (Hong Kong).

[18] Siebenrock KA, Unni KK, Rock MG (1998). Giant-cell tumor of bone metastasising to the lungs: a long-term follow-up. J Bone Joint Surg (Br).

[19] Averill RM, Smith RJ, Campbell CJ (1980). Giant cell tumors of the bones of the hand. J Hand Surg Am.

[20] Ropars M, Kaila R, Cannon SR, Briggs TW (2007). Primary giant cell tumours of the digital bones of the hand. J Hand Surg.

[21] Thomas D, Henshaw R, Skubitz K, Chawla S, Staddon A, Blay JY, Roudier M, Smith J, Ye Z, Sohn W, Dansey R, Jun S (2010). Denosumab in patients with giant-cell tumour of bone: an open-label, phase 2 study. Lancet Oncol.

[22] Werner M (2006). Giant cell tumour of bone: morphological, biological and histogenetical aspects. Int Orthop.

[23] Tian R, Su M, Tian Y, Li F, Li L, Kuang A (2009). Dual-time point PET/CT with F-18 FDG for the differentiation of malignant and benign bone lesions. Skeletal Radiol.

[24] Cowan RW, Singh G (2013). Giant cell tumor of bone: a basic science perspective. Bone.

[25] Vincenzo G, Vincenzo DL (1816). Role of p63 in cancer development. Biochim Biophys Acta.

[26] Babeto E, Luis A, Curado V, Peitl P, Zuccari D (2011). Differentially expressed genes in giant cell tumor of bone. Virchows Archiv.

[27] Lawson L, VanLerberg N, Tawfik O (1996). Pulmonary metastasis from a benign giant-cell tumor of the hand: report of a case diagnosed by fine-needle aspiration cytology. Diagn Cytopathol.

[28] Goldenberg RR, Campbell CJ, Bonfiglio M (1970). Giant-cell tumor of bone: an analysis of 218 cases. J Bone Joint Surg Am.

[29] Joly MA, Vazquez JJ, Martinez A, Guillen FJ (1984). Blood-borne spread of a benign giant cell tumor from the radius to the soft tissue of the hand. Cancer.

[30] Dominkus M, Ruggieri P, Bertoni F, Briccoli A, Picci P, Rocca M, Mercuri M (2006). Histologically verified lung metastases in benign giant cell tumours-1 cases from a single institution. Int Orthop.

[31] Jacopin S, Viehweger E, Glard Y, Launay F, Jouve JL, Bouvier C, Bollini G (2010). Fatal lung metastasis secondary to index finger giant cell tumor in an 8-year-old child. Orthop Traumatol Surg Res.

[32] Errani C, Ruggieri P, Asenzio MA, Toscano A, Colangeli S, Rimondi E, Rossi G, Longhi A, Mercuri M (2010). Giant cell tumor of the extremity: a review of 349 cases from a single institution. Cancer Treat Rev.

[33] Kremen TJ, Bernthal NM, Eckardt MA, Eckardt JJ (2012). Giant cell tumor of bone: are we stratifying results appropriately?. Clin Orthop Relat Res.

[34] Takeuchi A, Tsuchiya H, Niu X, Ueda T, Jeon D-G (2011). The prognostic factors of recurrent GCT: a cooperative study by the Eastern Asian Musculoskeletal Oncology Group. J Orthop Sci.

[35] Klenke FM, Wenger DE, Inwards CY, Rose PS, Sim FH (2011). Recurrent giant cell tumor of long bones analysis of surgical management. Clin Orthop Relat Res.

[36] Viswanathan S, Jambhekar NA (2010). Metastatic giant cell tumor of bone: are there associated factors and best treatment modalities?. Clin Orthop Relat Res.

[37] Balke M, Schremper L, Gebert C, Ahrens H, Streitbuerger A, Koehler G, Hardes J, Gosheger G (2008). Giant cell tumor of bone: treatment and outcome of 214 cases. J Cancer Res Clin Oncol.

[38] Donthineni R, Boriani L, Ofluoglu O, Bandiera S (2009). Metastatic behaviour of giant cell tumour of the spine. Int Orthop (SICOT).

[39] Faisham WI, Zulmi W, Halim AS, Biswal BM, Mutum SS, Ezane AM (2006). Aggressive giant cell tumour of bone. Singapore Med J.

[40] Prosser GH, Baloch KG, Tillman RM, Carter SR, Grimer RJ (2005). Does curettage without adjuvant therapy provide low recurrence rates in giant-cell tumors of bone?. Clin Orthop Relat Res.

[41] Su YP, Chen WM, Chen TH (2004). Giant-cell tumors of bone: an analysis of 87 cases. Int Orthop.

[42] Trieb K, Bitzan P, Lang S, Dominkus M, Kotz R (2001). Recurrence of curetted and bone-grafted giant-cell tumours with and without adjuvant phenol therapy. Eur J Surg Oncol.

[43] Blackley HR, Wunder JS, Davis AM, White LM, Kandel R, Bell RS (1999). Treatment of giant-cell tumors of long bones with curettage and bone-grafting. J Bone Joint Surg Am.

[44] O’Donnell RJ, Springfield DS, Motwani HK, Ready JE, Gebhardt MC, Mankin HJ (1994). Recurrence of giant-cell tumors of the long bones after curettage and packing with cement. J Bone Joint Surg Am.

[45] Kay RM, Eckardt JJ, Seeger LL, Mirra JM, Hak DJ (1994). Pulmonary metastasis of benign giant cell tumor of bone: six histologically confirmed cases, including one of spontaneous regression. Clin Orthop.

[46] McDonald DJ, Sim FH, McLeod RA, Dahlin DC (1986). Giant-cell tumor of bone. J Bone Joint Surg Am.

[47] Rock MG (1990). Curettage of giant-cell tumor of bone: factor influencing local recurrences and metastasis. Chir Organi Mov.

[48] Tubbs WS, Brown LR, Beabout JW, Rock MG, Unni KK (1992). Benign giant-cell tumor of bone with pulmonary metastases: clinical findings and radiologic appearance of metastases in 13 cases. AJR Am J Roentgenol.

[49] Kim Y, Nizami S, Goto H, Lee FY (2012). Modern interpretation of giant cell tumor of bone: predominantly osteoclastogenic stromal tumor. Clin Orthop Surg.

[50] Eckardt JJ, Grogan TJ (1986). Giant cell tumor of bone. Clin Orthop Relat Res.

[51] Faisham WI, Zulmi W, Mutum SS, Shuaib IL (2003). Natural history of giant cell tumour of the bone. Singapore Med J.

[52] Knochentumoren A (2008). Local recurrence of giant cell tumor of bone after intralesional treatment with and without adjuvant therapy. J Bone Joint Surg Am.

[53] Mendenhall WM, Zlotecki RA, Scarborough MT, Gibbs CP, Mendenhall NP (2006). Giant cell tumor of bone. Am J Clin Oncol.

[54] Wang HC, Chien SH, Lin GT (2005). Management of grade III giant cell tumors of bones. J Surg Oncol.

[55] Turcotte RE, Wunder JS, Isler MH, Bell RS, Schachar N, Masri BA, Moreau G, Davis AM (2002). Giant cell tumor of long bone: a Canadian Sarcoma Group study. Clin Orthop Relat Res.

[56] Malek F, Krueger P, Hatmi ZN, Malayeri AA, Faezipour H, O’Donnell RJ (2006). Local control of long bone giant cell tumour using curettage, burring and bone grafting without adjuvant therapy. Int Orthop.

[57] McGough RL, Rutledge J, Lewis VO, Lin PP, Yasko AW (2005). Impact severity of local recurrence in giant cell tumor of bone. Clin Orthop Relat Res.

[58] Campanacci M (1999). Bone and Soft Tissue Tumors.

[59] Tse LF, Wong KC, Kumta SM, Huang L, Chow TC, Griffith JF (2008). Bisphosphonates reduce local recurrence in extremity giant cell tumor of bone: a case-control study. Bone.

[60] Branstetter DG, Nelson SD, Manivel JC, Blay JY, Chawla S, Thomas DM, Jun S, Jacobs I (2012). Denosumab induces tumor reduction and bone formation in patients with giant-cell tumor of bone. Clin Cancer Res.

[61] Hamann C, Lützner J, Wieczorek K, Hofbauer LC (2012). Pulmonary metastases due to a giant-cell tumor of bone. J Clin Endocrinol Metab.

[62] Gupta R, Seethalakshmi V, Jambhekar NA, Prabhudesai S, Merchant N, Puri A, Agarwal M (2008). Clinicopathologic profile of 470 giant cell tumors of bone from a cancer hospital in western India. Ann Diagn Pathol.

